# A qualitative exploration of the sociocultural determinants of exclusive breastfeeding practices among rural mothers, North West Nigeria

**DOI:** 10.1186/s13006-019-0231-z

**Published:** 2019-08-20

**Authors:** Friday Ilop Joseph, Jane Earland

**Affiliations:** 1 Online Postgraduate Education, University of Liverpool, Liverpool, UK; 2 Department of Public Health and Policy, School of Medicine, Faculty of Health and Life Sciences, University of Liverpool, Liverpool, UK

**Keywords:** Exclusive breastfeeding, Qualitative, Perception, Sociocultural, North West Nigeria

## Abstract

**Background:**

Suboptimal breastfeeding is responsible for 96% of deaths among children under 12 months of age in developing countries. However, the exclusive breastfeeding rate in Nigeria from birth to 6 months is just 23%. The study explored the sociocultural factors that influence exclusive breastfeeding among rural mothers.

**Methods:**

The social constructionism-interpretivist epistemological approach underpinned this qualitative study. Semi-structured interviews were conducted with 20 mothers aged 18–39 years, purposefully sampled from two Local Government Areas in Katsina State, Nigeria. Thematic content approach was utilised for analysis.

**Results:**

Three major themes were developed from the analysis: (1) Breastfeeding initiation – the determinants of how soon a mother initiated breastfeeding included traditional new-born care practices, the birth attendant and place of delivery. (2) Exclusive breastfeeding - motivation to sustain exclusive breastfeeding was influenced by the conflict between the obligation to perform traditional rites, the mother’s awareness and family support. (3) Decision-making about infant feeding – the husband, grandmother, traditional birth attendant and the health workers all influenced participants’ decisions around infant feeding. Despite awareness of the benefits of exclusive breastfeeding among most mothers interviewed, they expressed concerns that they may not win their family’s support if their views were contrary to those held by other family members.

**Conclusion:**

While mothers have limited powers to make decisions, the key role that grandmothers and husbands have in decisions about breastfeeding demonstrates the need to engage the support of partners and relatives through community-driven policies and integrated interventions that address social and cultural barriers throughout the prenatal and postnatal period.

## Background

In 2015, the World Health Organization (WHO) estimated that 5.9 million under 5 year old children died from preventable diseases in Africa [1]. Suboptimal breastfeeding is responsible for 96% of deaths among children under 12 months of age in developing countries [2]. Two of the leading causes of infant and child mortality, namely pneumonia and diarrhoea, have been implicated in over 30% of under five deaths [3, 4]. Globally, half of all child deaths due to pneumonia and diarrhoea occurred in India, Nigeria, Democratic Republic of Congo and Ethiopia [3]. An infant not exclusively breastfed for 6 months is 15 times more likely to die from pneumonia and diarrhoea than exclusively breastfed children [1, 5]. Optimal breastfeeding is the most cost-effective child survival strategy [6] and has the potential to reduce under-five mortality by 13% in developing countries [5]. Optimal breastfeeding consists of early initiation within an hour of birth, exclusive breastfeeding from birth to 6 months of life and breastfeeding up to 2 years of age or beyond [7]. The WHO defined exclusive breastfeeding as an infant receiving only breast milk without food, drink, and water. Human breast milk provides all the energy, nutrients and fluid an infant needs for optimal growth and development in the first 6 months. It provides natural passive immunization that greatly reduces the risk of an infant developing respiratory infections and diarrhoeal disease [8, 9]. Despite the benefits of optimal breastfeeding practices, in Nigeria, the 2016–2017 Multiple Indicator Cluster Survey (MICS) indicated that only 23% of infants below 6 months of age were breastfed exclusively [10]. The estimated population of Nigeria is over 180 million, and the Infant Mortality Rate is 70 per 1000 live births [10]. Although, Nigeria’s infant mortality rate is ranked sixth in Africa, there are more infant and under five deaths in Nigeria than any country in Africa [11, 12].

In its 2015 ‘Breastfeeding policy brief’ WHO stated that social and cultural factors were among the six identified determinants of low exclusive breastfeeding rates globally. In a review of literature on barriers to optimal breastfeeding practices in West Africa, Apanga reported sociocultural practices, traditional beliefs and pressures from families as major barriers [13]. It was also noted that some women in South West Nigeria avoided exclusive breastfeeding to prevent sagging of the breast and ensure they remain attractive to their partners [13]. Additionally, the level of education of women in Nigeria influenced their decision to breastfeed exclusively, with those attaining a higher level of education more likely to exclusively breastfeed. The review also showed that mothers who had a hospital delivery were more likely to breastfeed their infant than their counterpart that had a home delivery [13]. Generally, studies across African countries have revealed that the delay in the initiation of breastfeeding is due to discarding colostrum and traditional rites performed on mother and baby [14–38]. There are also gender difference in the initiation of breastfeeding, with breastfeeding usually delayed for 3 days for a male child and 4 days for a female child [14–16, 20]. According to the studies reviewed, mothers hold a number of beliefs about breastfeeding including that first milk (colostrum) is bad for the child, water should be given to quench their thirst and rituals prior to the initiation of breastfeeding need to be performed to ensure that the breast is free of poisons [14, 15, 26, 38]. Also, most mothers, their partner and grandmothers (of the child, so the mother’s own mother or her mother-in-law) are of the view that breast milk is not adequate alone for the first 6 months [16–19, 34, 38]. Grandmothers and traditional birth attendants (TBA) play important and sometimes coercive roles in the initiation of breastfeeding [16, 17, 20], the practice of exclusive breastfeeding [21, 26, 32, 33] and discarding colostrum [14, 20].

Given the lack of qualitative research studies on breastfeeding practices in the study area and the importance of exclusive breastfeeding in reducing infant mortality and morbidity, this study explored the sociocultural factors that affect a mother’s choice to commence and sustain exclusive breastfeeding for 6 months in North West Nigeria. It is hoped that the findings will inform future programmes in Nigeria designed to increase the rate of exclusive breastfeeding in the first 6 months, towards the global nutrition target of at least 50% by 2025 [39].

## Methods

The social constructionism-interpretivist epistemological approach underpinned the design of this qualitative study [40]. The study explored the sociocultural beliefs and practices of rural mothers that influenced the timely initiation and practice of breastfeeding. What informed the choice of this approach was that people’s actions and attitudes cannot be separated from their shared assumption and beliefs [41, 42]. In-depth individual interviews were conducted which allowed a deeper understanding of participants’ thoughts and practices through their shared experience on breastfeeding.

The study was carried out in Bindawa and Baure Local Government Areas in Katsina, a state in North West Nigeria. Over two million people live in the study area, comprising Hausa and Fulani Muslims. The Fulani are nomads while the Hausa are agrarians. The National Bureau of Statistics [43] reported in 2015 that 78.4% of women in Katsina state had no formal education, while 84.4% can neither read nor write. In Katsina state, 38% of women initiated breastfeeding within 1 h after delivery, 6.7% of infants between 0 and 5 months were exclusively breastfed and the median duration of exclusive breastfeeding was 0.4 months [10].

Purposive sampling was used to recruit an information rich sample [40]. The inclusion criteria were that participants should be mothers within the age range of 18 to 45 years with at least one child which was breastfeeding at the time of the study or which had been breastfed within the previous 5 years. Mothers who were ill or pregnant were excluded from the study. The study participants were recruited from rural public primary health centres. The researcher discussed the research and approval for the study with the Primary Health Care Directors responsible for the primary health centres in these sites who informed the heads of the primary health centres about the study. The primary health care facility provides weekly maternal and child health outpatient interventions in the rural communities. The first author, accompanied by a translator, approached a total of 28 mothers during the routine clinic visits and introduced the research using the participant information sheet. The participant information sheets were written in Hausa but as the majority of rural mothers are illiterate the purpose of the study, the inclusion and exclusion criteria, confidentiality, anonymity and voluntary participation were discussed in Hausa with the potential participants [44, 45]. Mothers took the participant information sheet home to discuss with their husbands who, given that this is a male dominated society, would likely need to have given approval for their wives to participate in the study. The researcher visited the primary health centres during their next clinic visits and approached the same mothers with the translator who, through the researcher, answered mothers’ concerns and queries. Only 23 mothers, all of whom met the inclusion criteria, attended the next clinic. All volunteered to participate. Although it is possible that the remaining five did not wish to take part in the study, non-attendance may be due to a number of reasons. The clinic setting was identified independently by the participants as the most appropriate place for the interview and suitable dates were agreed upon. The interviews were conducted in a private room at the clinics between June and July 2016. Although 23 mothers were interviewed, only the interviews from 20 mothers were transcribed and analysed. The other three mothers confirmed to the researcher that their responses had been influenced by a discussion held with a volunteer community nutrition assistant prior to the interviews so it was decided to exclude their interviews from the study. Although five participants were Fulani, with the remainder Hausa, all could understand and communicate in Hausa.

The participants were aged between 18 and 39 years, with the majority 22–35 years. Only four participants had received some education, at Arabic School. Islam is the religion practiced by the participants of the study. The participants’ sociodemographic data are shown in Table 1.

**Table 1:** Participants’ sociodemographic information

Characteristics	n (*N* = 20)
Ethnicity	
Hausa	15
Fulani	5
Education	
Formal education	Nil
Informal education (Arabic school)^a^	4
No education	16
Occupation	
Housewife	19
Subsistence farmer	1
Place of last birth	
Home	17
Health centre^b^	3
Number of antenatal clinic visits	
3 or more visits	6
1–2 visits	7
No visits	7

^a^Those Arabic schools not recognized by the Government are referred to as informal

^b^One participant had a caesarean birth

Data were collected using a semi-structured interview guide. The key questions covered the participants’ experience within the first hour of delivery, initiation of infant feeding, feeding practices from birth to 6 months, mothers’ understanding about exclusive breastfeeding and what determines a mother’s choice to breastfeed exclusively. Probes were used to enable participants to provide a rich account of their experience [46, 47]. Two pilot interviews were conducted with participants that met the inclusion criteria. The quality of the pilot interviews was reviewed by an experienced qualitative researcher (the second author). As only minor adjustments were made to the interview guide, the pilot transcripts were included in the analysis. The interviews were conducted in English and Hausa, with the female translator translating each communication between the researcher and participants. Audio recorders were used to record the interviews.

The interviews were transcribed in Hausa before translation into English by the researcher, with the support of the translator. The data were analysed manually using thematic content analysis [48, 49]. The researcher coded each transcript by reading it through several times and attaching words or a phrase to form a code to describe each statement or new issue raised by the participant. Following this initial coding, each transcript was compared against other transcripts noting patterns of similarities and differences. The final codes were formed by either merging or dividing the initial codes and renaming, if necessary [50]. Subthemes were formed by grouping related codes. Similarly, related subthemes were pooled together to form the major themes. The analysis was a continuous process until the researchers judged that the final codes, themes and subthemes fully captured the participants’ accounts. Thus the themes and subthemes were not predetermined but were derived inductively during the analysis of the data [51].

## Results

Three main themes were derived from the analysis: Breastfeeding initiation, Exclusive breastfeeding and Decision-making on infant feeding. The six subthemes included participants’ personal experiences of infant feeding, determinants of how soon breastfeeding starts, and social and cultural influences on participants’ choice and practices of exclusive breastfeeding.

### Theme 1: breastfeeding initiation

A few mothers met the WHO recommendation of initiating breastfeeding within 30 min to 1 h after delivery. The delays were, in most cases, due to postnatal traditional and religious rites.

#### Personal experience of infant feeding

All except two participants noted that immediate new-born traditional practices differ between the first delivery and subsequent births. New mothers wait for 3 days before breastfeeding a male child and 4 days for a female baby. Herbs were used to treat the mother’s breast after 2 days in preparation for breastfeeding. The majority of the multi-parous participants independently confirmed the response of the three first-time mothers in the study when they highlighted their experience of their first delivery:

*“ … If it was my first birth that is when I was told what to do; if it’s a male three days before breastfeeding the baby while if it’s a female its four days before breastfeeding the baby” [Participant 1, 30 years].*

*“Before they were saying that I must spend three days before breastfeeding, and the child was crying and if we give him goat milk” [Participant 2, 30 years].*

There are alternative feeding methods which participants explored during the period of delayed initiation of breastfeeding. Most participants fed their infants with either goat’s or cow’s milk in addition to dates, Islamic water (water which has been blessed by a cleric), traditional herbs or honey. Most of the participants reported that a new mother routinely receives instruction on the care of the new-born from the grandmother or TBA.

*“That is what we know, it is the tradition, and if we deliver before we give anything they [mother-in-law and TBA] say that we should give dates, holy water and honey. We did not do anything with it [colostrum]. After they gave the baby dates and holy water I started breastfeeding the baby” [Participant 3, 29 years].*

During the period before the initiation of breastfeeding, only among the Fulanis was it a common practice to use a wet nurse.

*“So, how many days did you stay before breastfeeding? [interviewer] … two days, she was breastfed by a different mother, my younger sister breastfed my baby … She breastfeeds my baby before they expressed my own breast milk” [Participant 16, 35 years].*

#### Colostrum

Referring to their first birth, the majority of participants discarded colostrum. The view of these mothers was that colostrum is dirty and a baby can contract diseases from it. A few took herbs to make the perceived bad milk good for the baby.

*“You know we are in the village, some will say something [about disease or infection]. The fear that the child might encounter something. You know that time we don’t want the baby to contact disease from the first milk [colostrum]” [Participant 18, 30 years].*

*“So, during this period [delayed initiation of breastfeeding], what did they [attendants] do to your breast milk?” [Interviewer] “ . . . they brought the herbs and gave me to drink and they give to the baby . . .” “What is the function of the herbs?” [Interviewer] “It makes the breast milk to be pure” [Participant 8, 30 years].*

This Fulani mother held the view that rubbing a mixture of herbs and cheese on the breast makes the breast milk good:

*“. . . [laughing] oil gotten from cow milk [cheese], and they mix it with a traditional herb, and then rub it on the breast that’s all [laughing] . . . It makes the breast milk good . . .” [Participant 16, 35 years].*

While a new mother’s breast milk must be ‘checked’ by a grandmother or a TBA before breastfeeding, in subsequent deliveries colostrum was not discarded. After mother and baby are washed, mothers just washed their breast with herbs, and breastfed their baby:

*“ . . . It is only for the first pregnancy they do that [check whether the milk is good by dropping the breast milk on a flat metal surface of a hoe that is slightly titled at some degree above the ground surface; if it flows down it is good] . . . You just wash the breast with the traditional medicine that is all” (Participant 13, 21 years].*

A few participants neither discarded colostrum nor took traditional herbs, irrespective of whether it was the first or subsequent deliveries because it was not the tradition in the family to give local herbs and they were counselled that colostrum is good for baby during the routine antenatal clinic.

*“We did not give him anything . . . In our house immediately after delivery we breastfeed the baby . . . I was informed in the hospital, when we came for antenatal clinic they said that we should breastfeed our children from birth to six months, only breast milk.” [Participant 20, 35 years].*

#### Factors that determine how soon breastfeeding starts

The time of the first feed was influenced by a number of traditional new-born care activities; the place of delivery, practices of the birth attendant, family response, religious beliefs, and health seeking behaviour.

*“ . . . She [TBA] cut the umbilical cord, after she cut the cord we warm water . . . they brought the water to her and she bath the baby . . . She bath the baby, and also washed the placenta, then she gave me the baby and left” [Participant 9, 30 years].*

Bathing of mother and baby before initiating breastfeeding was a very common practice among participants. Even though the majority of participants went for at least two antenatal clinic visits before delivery, the majority had a home delivery. Participants that had a hospital delivery breastfed their babies earlier than those that had a home delivery.

*“ . . . they [mother-in-law and relatives around me] said that I must bath before breastfeeding the baby, but in the hospital, they will just ask you to breastfeed the baby” [Participant 20, 35 years].*

*“ . . . Immediately after birth I took my bath and they also bath the baby, and I started breastfeeding the baby . . . ” [Participant 7, 22 years].*

While most of the participants received health education on optimal infant feeding during the antenatal clinic visits, they were frightened of how their family would respond to their optimal infant feeding practice.

*“You know it is first delivery you do what they [elderly mothers] ask you to do. They said that we must wash the breast and express the breast milk out before giving to the baby” [participant 16, 35 years].*

The majority of participants gave pre-lacteal food and fluid – holy water, dates, honey, herbs, for either traditional or religious reasons, which delayed the onset of breastfeeding.

*“ . . . we gave the baby a drop of Islamic medicine [Islamic cleric write koranic words on a slate, then washes the writing into a cup] just small amount, then we rub the baby body with it. We rubbed the baby with the Islamic medicine, before I started breastfeeding the baby” [Participant 12, 30 years].*

The way a few participants managed commencement of breastfeeding during maternal illness or breastfeeding problems indicates a poor understanding by health workers on how to manage common breastfeeding problems.

*“I delivered on Monday and they transfuse one bag of blood to me, and added another bag on Tuesday . . . I breastfeed her the first time and when I was having bleeding, I did not breastfeed her again until in the morning” [Participant 14, 18 years, first time mother].*

### Theme 2: exclusive breastfeeding

Participants discussed where they obtained information on infant feeding and the challenges of sustaining exclusive breastfeeding. While inadequate family support, uvulectomy and participants’ impression that break milk is inadequate for a baby were barriers, partners’ awareness and views on the benefits of exclusive breastfeeding were a facilitator to exclusive breastfeeding.

#### Information on infant feeding

The majority gave an accurate definition of exclusive breast feeding as infant feeding with breast milk alone from birth to 6 months.

*“They [health workers] normally told us that, it is good to breastfeed exclusively, that mothers should stop giving water, cow milk and pap to their babies if the baby is not up to six months . . . ” [Participant 11, 25 years].*

Several participants noted that health workers were the source of information on optimal infant feeding during their routine clinic visit. Two participants that did not go to the antenatal clinic received information on infant feeding from community members.

*“ . . . she [TBA] told me to take care of the baby, and I should not give the baby anything apart from breast milk . . . She heard it from her friend who is a traditional birth attendant . . .” [Participant 12, 30 years].*

#### Factors influencing the sustainability of exclusive breastfeeding

Participants noted a number of factors that determined whether they continued to breastfeed for the recommended 6 months. The majority identified two cultural practices which were prominent in their areas: traditional uvulectomy on the baby and 40 days of traditional bathing for mothers. Following an uvulectomy, some infants develop an infection that interrupts breastfeeding while the mother focuses on treating the baby with oral herbal medication. When probed for the reasons for some practices, no specific reasons were given and the practices were ‘just tradition’ or they were just told to do it. In contrast, a few participants reported they never practise uvulectomy as it is not a family practice:

*“Did you remove the uvula?” [Interviewer]. “. . . yes, they [traditional barber) remove the uvula”. “What are the reasons for removing the uvula?” [Interviewer] “. . . They just told us that it is tradition” [Participant 8, 30 years].*

*“In his father’s family house, when a woman gave birth, they don't remove it . . .” [Participant 11, 25 years].*

Another common practice highlighted by most participants was the 40 days of traditional bathing of the mother.

*“During that time [40 days of traditional bathing] I don't even work at home; most of my work at home was done by someone else, and I am always with the child and I was breastfeeding” [Participant 4, 39 years].*

Only a few mothers and their families believed that breast milk alone provides sufficient fluid for an infant.

*“They [health workers] told us that there is water in breast milk, and if we want to see it, we should express the breast milk in a cup and leave it for some time, you will see the water settled down and we believe them” [Participant 4, 39 years].*

Despite adequate breastfeeding awareness among most participants, there were personal opposing views about the adequacy of breast milk alone for an infant.

*“. . . if we adult did not drink water we will be thirsty, now if I did not give him water he used to cry. Yes, I see that the water in the breast milk will not quench his thirst . . .” [Participant 3, 29 years].*

Support by family members and participants’ knowledge of the benefits of exclusive breastfeeding were strong motivational factors to the few participants that practised exclusive breastfeeding. These mothers did not undergo traditional rites beyond washing the mother and the baby.

### Theme 3: decision-making about infant feeding

A number of social figures influence a mother’s ability to make decisions about infant feeding, to varying degrees. The most influential ones are their partner, grandmother, TBA and health workers. However the quality and degree of influence these figures have depend on the person’s exposure to breastfeeding promotion messages, their position in social hierarchy in relation to the mother and how much time they spend with the mother.

#### Influence of family members and the TBA

Although husbands are often away from the family because of their work, which limits their participation in the day-to-day care of the infant, they have more influence than the grandmother.

*“Now it is a new generation, once if his father agree [that his child should be exclusively breastfed] that is all, because he has all right to do to his child what he thinks is good.” [Participant 12, 30 years].*

Traditionally, the mother-in-law provides their son’s wife with support in the daily care of their grandchildren. As they are always with their son’s wife, they give instructions to the mother on caring for the infant.

*“. . . But you know they said we should drink, I will not say that I don’t want to drink it. They [mother-in-law and TBA] said it [herbs] should be boil and given to her [baby]. Since the traditional birth attendant say that I should give her, I did not ask, we just boiled the herb and gave to her” [Participant 8, 30 years].*

However, in a situation where the husband’s decision contradicts the mother-in-law, his decision prevails due to the patriarchal nature of a typical African society. Where the grandmother is not the TBA, the role of the latter is limited to the immediate traditional postnatal new-born care.

*“. . . here the old women do that, we are in the village, but now in the urban areas some of the women cut the cord themselves, but here in the village we call the traditional birth attendant to do that.” [Participant 1, 30 years].*

#### Influence of health workers on infant feeding practices

Health workers are a potential source of information on appropriate infant feeding practices. However, as only a few mothers attended antenatal clinic more than twice throughout their pregnancy and the majority had a home delivery, they had limited contact time with the health worker compared to their mother or mother-in-law. However, advice given by health workers was seen as the most credible by a few husbands.

*“. . . When I told my husband what the health workers say about exclusive breastfeeding he [husband] said it was ok, he gave me the permission to go ahead . . .” [Participant 11, 25 years].*

*“I was two month pregnant when he [husband] sent me to the nearest hospital to register for antenatal care and they told us there that if we give birth we shouldn’t give any other thing apart from breast milk, then they weren’t saying until six months but till the child start to sit, you know when a child is able to sit down and hold something he/she may be able to feed himself or herself” [Participant 4, 39 years].*

Seeing that exclusively breastfed infants were healthy also influenced the husband of one participant to follow the advice of the health worker:

*I was informed in the clinic, when we came for antenatal care they [health workers] said that we should breastfeed our children from birth to six month, only breast milk . . . but my mother-in-law said that I should give water, and his father said until he is six month . . . what informed my husband’s decision is my husband’s elder brother [who worked in a health clinic]; his wives are practising exclusive breastfeeding and he saw that his children are healthy and okay, that is why he said my children should not be given water.” [Participant 20, 35 years].*

## Discussion

The findings from this study revealed a number of harmful sociocultural practices that delayed breastfeeding initiation and prevented exclusive breastfeeding from birth. Consistent with other studies conducted in Ethiopia [22, 23, 29, 30], Ghana [15, 16], South Africa [21] and Tanzania [38], most participants’ personal accounts revealed the use of prelacteal fluid and food before the initiation of breastfeeding, which could sometimes continue until 4 days after birth, irrespective of the whether it was the first or subsequent deliveries. Although the participants in the present study identified that the intended purpose for prelacteal feeding was to protect the baby, prelacteal fluids are a predisposing factor to diarrhoea among new-borns [52–54]. The potential consequence of this practice is the impact on diarrhoea specific infant death in developing countries like Nigeria [55, 56]. Participants in the present study shared religious beliefs which influenced their actions. Most reported that either the mother, husband or grandmother were instructed by the religious leaders to give a solution made of Islamic writing, dates and honey, as well as holy water. There were similar findings from a survey in north west Nigeria, where mothers considered colostrum spoilt and fed their babies with either animal milk, honey or washouts from writings of the Koran on slates while awaiting the coming of the clean, unspoilt milk [37]. While it appears from the literature review that there are no studies on the role of religious leaders in promoting exclusive breastfeeding, Aborigo et al. identified the need to involve religious leaders in future interventions to promote optimum infant feeding practices [15]. Their qualitative study in Ghana showed an association between religious beliefs and compliance with breastfeeding guidelines; specifically Christians adhered to the guidelines in contrast to traditionalists who preferred to follow traditional practices. These findings may not be transferable to the setting of the present study where the participants and population practise Islam. Given the importance of religious practices in relation to breastfeeding shown in this and many studies [15, 37], there is clearly a need for further exploratory research with religious leaders to understand how best to engage these stakeholders in community breastfeeding promotion activities.

The belief most mothers held that colostrum is harmful to the baby was also shown in previous qualitative research conducted in Ethiopia [14, 20]. However, it was not clear in the literature if the colostrum is discarded only after the first delivery or also after subsequent births. In this study participants reported that the practice of discarding colostrum was only practised among first time mothers. Very similar to the findings in a study by Legesse, Demena, Mesfin and Haile [14] on colostrum avoidance in Ethiopia, one participant described how the mother-in-law and TBA enforced the practice of discarding colostrum. In most cases the TBA is the oldest mother in an extended family with no formal health training. This and other findings which demonstrated the influence of grandmothers and TBA on infant feeding decisions buttressed influential females in rural communities as potential targets for health promotion and educational messages. While participants of the Hausa ethnic group gave other forms of milk during the period before breastfeeding was initiated (3 days for male babies and 4 days for female babies), two Fulani participants used a close relative as a wet nurse. The use of a wet nurse where there are contraindications to breastfeeding has been found in other studies [15, 34]. As this study’s focus was not on the use of a wet nurse as an alternate to the mother breastfeeding, the researchers did not explore this further, though it appears to be an interesting area for future research.

Birth attendants, place of delivery, and the health status of the mother after delivery have been shown in other studies to be determinants of timely breastfeeding initiation [16, 22]. However the findings from this study indicated that the knowledge and perceptions of the birth attendant who assisted the mother during childbirth (either the TBA or a nurse if the infant is delivered in a health facility) are also crucial, regardless of where the delivery took place. One participant stated that although she had a home delivery, the trained TBA helped her to initiate breastfeeding soon after birth. The majority of participants were attended by a TBA during their home delivery. Two of these were trained by international Non-Governmental Organizations on basic aseptic techniques for child delivery in rural settings. The WHO does not advocate the use of TBA for community childbirth, due to conflicting evidence on their effectiveness in reducing childbirth related maternal mortality [57, 58]. However, where the majority of deliveries are not in a health facility and where TBA are widely used, as in this study area, there are good prospects for the trained TBA to promote community infant feeding and breastfeeding, acting as a linkage between the community and health facility. Timely breastfeeding initiation has been reported among infants where mothers were assisted by trained TBA even though these mothers had a home delivery [22, 59]. It was also reported in another study in Tanzania that most registered TBA recognised that a health centre is the best and safest place for the handling of pregnancy complications even though most participants in the study had a home delivery [60]. It is worth noting that most participants, and likely their families, in this study chose a home birth in spite of attending an antenatal clinic prior to delivery. The reasons for choosing a home birth was not explored as it did not fall within the scope of the study; however this is an area of great concern in regards to maternal and new-born health in Africa. A study in Tanzania found that mothers chose a home birth due to various factors including distance to the health facility, past experience of a safe delivery at home, cost of a hospital delivery, health workers’ poor attitudes or lack of hospital bed space [60].

It was also reported in this study that while a few heard about exclusive breastfeeding at community gatherings such as weddings or child-naming ceremonies and from role models, health workers continued to be the main source of breastfeeding information during the antenatal clinic visit for the majority of participants. This finding on the key role of health workers in providing information on breastfeeding was corroborated by several studies that explored the role of health workers in breastfeeding promotion [15–17, 20, 22, 24, 25, 31, 35]. However a number of studies have highlighted barriers to infant and young child feeding education being provided by health professionals including poor organization and inadequate or inaccurate information on breastfeeding [17], high workload and insufficient staff [16, 35]. Therefore, health workers need to be supported in their health promotion role as they perform multiple tasks. Many working in rural areas are inadequately skilled due to limited opportunities to improve their medical knowledge and skills [38].

Similar to findings from other studies [13, 15, 17, 26, 34–36, 38], the belief amongst most participants and other members of the community that breast milk alone is not an adequate source of fluid for the infant due to the arid environment was another barrier to exclusive breastfeeding. The participants’ accounts revealed other harmful sociocultural practices that may not have supported exclusive breastfeeding among these mothers. The majority described the practice of infant uvulectomy by a traditional barber around the seventh day of life due to a belief that the uvula grows and kills the baby if not removed. In most cases the procedures are not aseptic with the probability of post-procedure infection and delayed breastfeeding. One of the participants reported recurrent post-uvulectomy emesis. This is an important finding which has not been reported in other breastfeeding studies. This may have been because in other studies the researchers did not consider the impact of uvulectomy on infant feeding or participants knew the practice was harmful and illegal in many countries and therefore did not reveal it. Removal of babies’ uvula indicates the need for behavioural change messages to dissuade the community from harmful traditional practices. Forty days of regular bathing and seclusion of the mother and baby was another common practice among participant in this study; a similar finding was reported in other studies among rural Kenyan [33] and peri-urban Ghanaian mothers [17]. Mothers are assisted by family members to do household chores during this period. This practice appears to be an opportunity to promote optimal breastfeeding practice and for the mother to rest and therefore should not be discouraged.

A number of studies have shown a direct relationship between antenatal and postnatal breastfeeding education on early initiation and exclusive breastfeeding rates [18, 24, 27, 35, 36, 38]. In this study, most mothers received counselling on exclusive breastfeeding but did not practice it. It has been shown elsewhere that awareness of optimal breastfeeding messages does not necessarily translate into practice [15]; this situation indicates that there are other confounding social factors which may influence a mother’s decision to exclusively breastfeed [35]. A typical African family is male-controlled, so the husband is seen as the head of the family and makes most decisions that relates to the nuclear family. However this may not traditionally include breastfeeding and caring for the new-born baby, including rituals following the birth, which are seen as the responsibility of the mother and her mother or mother-in-law or the TBA [61]. It may be possible to change traditions such as these. Focus group discussions with fathers in Ghana revealed that there is the potential and willingness for fathers to be more involved in decisions on infant feeding, including exclusive breastfeeding [61]. While there are no in-depth studies on males’ perceptions on their roles in promoting breastfeeding, Seid, Yesuf and Koye [25] reported a direct relationship between paternal education and mothers’ optimal breastfeeding practices, which implies that male partners adequately exposed to the value and importance of optimal infant feeding can positively influence breastfeeding practices. In this study, one participant exclusively breastfed her baby for 4 months with approval from her husband to practice what was recommended during the antenatal clinic visits, which underscores the importance of including males in strategies to promote exclusive breastfeeding. It is a custom in many African countries that after delivery, the mother-in-law stays with the mother for a least 1 month to provide support for the mother and new-born, while the husband is away from the house for routine business [14, 15, 30]. Although most of the participants in the present study were aware of exclusive breastfeeding, they could not make informed decisions about feeding their baby, as their choice would be overturned by the mother-in-law. Legesse et al. [14] reported that the most influential figures in the discarding of colostrum are grandmothers (44%), untrained TBAs (44%) and husbands (12%). Similar studies [14, 15, 17, 20, 29, 35, 38] have corroborated this finding that grandmothers, especially the mother-in-law, and untrained TBAs usually enforce suboptimal breastfeeding practices.

The limitations of the study included recall bias; a few participants could not recall some of their experiences which may have limited how much information they shared. Recruiting mothers whose youngest infant was less than 12 months would have addressed this potential limitation. Some participants may have found it difficult to discuss personal issues with a male, although the female translator was present at all of the interviews. Triangulation of multiple sources like the mother-in-law, TBA and father may have strengthened the evidence in this study, although their perspectives may have been different to those of the participants. Interview transcripts and the analysis were reviewed by the second author who is an experienced qualitative researcher, familiar with the subject of enquiry. Frequent debriefing sessions with the translator who had qualitative research experience on infant feeding helped the researchers to mitigate the effects of their positionality [62] and views through the course of the interviews. This and the other strategies used helped to enhance the rigour of data collection, analysis and interpretation [63–66].

## Conclusion

The study revealed that the birth attendant, family and traditional practices affect optimal breastfeeding practices. It also highlighted the fact that mothers have limited powers to make decisions. There is therefore a need for culture-sensitive and community-driven policies and integrated interventions throughout the prenatal to postnatal period that address social and cultural barriers by engaging and empowering the community. The findings from this study, including the key role that grandmothers and husbands have in decisions about breastfeeding, demonstrate the need to engage partner and relative support and commitments to exclusive breastfeeding. Further exploratory research is necessary to understand the experience and views of mothers who had delivered in a health facility as well as the views of other community members such as religious leaders; it would enable the development of programmes which involve all sections of the population which influence infant feeding practices.

## Data Availability

The datasets used and/or analysed during the current study are available from the corresponding author on reasonable request.
